# Output-Only Damage Detection in Plate-Like Structures Based on Proportional Strain Flexibility Matrix

**DOI:** 10.3390/s20236862

**Published:** 2020-11-30

**Authors:** Kang Yun, Mingyao Liu, Jiangtao Lv, Jingliang Wang, Zhao Li, Han Song

**Affiliations:** 1 School of Mechanical and Electronic Engineering, Wuhan University of Technology, Wuhan 430070, China; yunkang@whut.edu.cn (K.Y.); ljt266828@163.com (J.L.); wangjl0395@whut.edu.cn (J.W.); lizhao2017@whut.edu.cn (Z.L.); songhan@whut.edu.cn (H.S.); 2 Hubei Key Laboratory of Digital Manufacturing, Wuhan University of Technology, Wuhan 430070, China

**Keywords:** damage detection, strain flexibility matrix, plate-like structures, structural health monitoring, uniform load strain field

## Abstract

For engineering structures, strain flexibility-based approaches have been widely used for structural health monitoring purposes with prominent advantages. However, the applicability and robustness of the method need to be further improved. In this paper, a novel damage index based on differences in uniform load strain field (ULSF) is developed for plate-like structures. When estimating ULSF, the strain flexibility matrix (SFM) based on mass-normalized strain mode shapes (SMSs) is needed. However, the mass-normalized strain mode shapes (SMSs) are complicated and difficult to obtain when the input, i.e., the excitation, is unknown. To address this issue, the proportional strain flexibility matrix (PSFM) and its simplified construction procedure are proposed and integrated into the frames of ULSF, which can be easily obtained when the input is unknown. The identification accuracy of the method under the damage with different locations and degrees is validated by the numerical examples and experimental examples. Both the numerical and experimental results demonstrate that the proposed method provides a reliable tool for output-only damage detection of plate-like structures without estimating the mass-normalized strain mode shapes (SMSs).

## 1. Introduction

Damage detection is a crucial component of structural health monitoring (SHM) systems. Significant strides have been made in damage detection methods for developing a reliable SHM system [1]. Most of these methods are based on the vibration property, i.e., modal parameters. A comprehensive review of modal parameter-based damage identification methods for the engineering structure was presented in [2], and the damage identification algorithms in terms of signal processing were particularly emphasized.

The damage detection methods, based on the measured modal parameters, can be classified as model-based methods and non-model-based methods [3]. Generally, the model-based methods [4,5] refers to the model updating method, in which different nonlinear optimization methods are used to update the theoretical model, i.e., the finite element model (FEM) or the analytical model, close to the experimental model. Thus, it is crucial to construct an ideal FEM that can substitute the actual situation of the structure to improve the effectiveness of those methods. Due to the simplified modeling assumptions, inaccurate boundary conditions, material uniformity assumption, and other sources of errors, the development of an initial FEM of a structure often differs from the actual structure.

Non-model-based damage detection methods are relatively straightforward when compared with the model-based damage detection methods. Changes in modal parameters between the intact state and the damaged state of the structure are usually used directly or in association with other relevant information to form damage indexes for damage detection purposes. Some damage indexes completely rely on the shift in natural frequencies [6,7], displacement mode shapes [8,9] and strain mode shapes (SMSs) [10]. To enhance or extend these methods, some researchers use the derivative of natural frequencies [11] and mode shapes [12], and integrate them to form some new damage indexes (i.e., modal assurance criteria [13], coordinate modal assurance criteria [14], and modal strain energy [15,16]). Moreover, the subspace-based damage indicators are also employed to conduct the damage detection [17,18].

Over the past decades, some researchers found that flexibility, especially strain flexibility, can be a sensitive damage index for SHM systems. Zhao and Dewolf confirmed that modal flexibility is more sensitive to damage than either the modal shapes or the natural frequencies through a theoretical sensitivity study [19]. Hong et al. proved that the modal strain is more sensitive to local changes of structure than displacement modal [20]. Zonta et al. reformed the flexibility matrix in a strain coordinate system to obtain strain flexibility matrix (SFM) [21]; numerical and experimental examples demonstrated that the strain flexibility is more sensitive to damage compared with displacement flexibility.

It should be noted that SFM can be estimated only when mass-normalized SMSs are available [22,23] (for example, in the input–output vibration test, at least one actuator–sensor pair is known). However, it is considerably difficult to measure the input signal in an actual working environment. To date, SFMs are not readily available when only arbitrary-scaled SMSs are obtained. In the present study, a proportional strain flexibility matrix (PSFM) from arbitrary-scaled SMSs and natural frequencies with output-only dynamic strain information is proposed. This feature means that the proposed method can be carried out the under operational conditions. Moreover, the uniform load surface (ULS) method proposed by Zhang and Aktan for damage localization is developed to the strain modal space by integrating the proposed PSFM into the frames of the defined uniform load strain field (ULSF) [24].

The remainder of the paper is organized as follows: Section 2 provides the definition of PSFM and damage index; the improved method for constructing the PSFM is shown in Section 3; Section 4 displays the results and discussions; Section 5 presents the conclusion.

## 2. Definition of PSFM and Damage Index

### 2.1. Definition of PSFM

For an *n*-degrees of freedom (DsOF) structural system, the classical SFM $F^{\varepsilon }$ can be constructed from a mass-normalized SMS vector ${\bar{\psi }}_{i}^{\varepsilon }$ and circular natural frequencies ${\bar{\omega }}_{i}$ as follows:

(1) $$F^{\varepsilon }=\underset{i=1}{\overset{n}{\sum }}\frac{1}{{\bar{\omega }}_{i}^{2}}{\bar{\psi }}_{i}^{\varepsilon }{\left({\bar{\psi }}_{i}^{\varepsilon }\right)}^{T}\;$$

where *T* means transpose operation. Estimating SFM through Equation (1) is not feasible in the condition of output-only SHM, where arbitrary-scaled SMSs rather than mass-normalized ones are available.

In present study, the *i*th arbitrary-scaled SMS $\psi_{i}^{\varepsilon }$ is defined as follows:

(2) $$\psi_{i}^{\varepsilon }=r_{i}{\bar{\psi }}_{i}^{\varepsilon }$$

where $r_{i}$ is the mass normalization factor for $\psi_{i}^{\varepsilon }$. Combining Equation (1) with Equation (2), one can obtain:

(3) $$F^{\varepsilon }=\underset{i=1}{\overset{n}{\sum }}\frac{1}{{\left(r_{i}{\bar{\omega }}_{i}\right)}^{2}}\psi_{i}^{\varepsilon }{\left(\psi_{i}^{\varepsilon }\right)}^{T}=\underset{i=1}{\overset{n}{\sum }}\frac{1}{\omega_{i}^{2}}\psi_{i}^{\varepsilon }{\left(\psi_{i}^{\varepsilon }\right)}^{T}$$

in which

(4) $$\omega_{i}=r_{i}{\bar{\omega }}_{i}$$

Comparing Equation (3) with Equation (1), $\psi_{i}^{\varepsilon }$ can be defined as the *i*th mass-normalized SMS pertaining to the natural frequency $\omega_{i}$,—the same as the definition of ${\bar{\psi }}_{i}^{\varepsilon }$.

The SFMs in Equations (1) and (3) can be regarded as two structures, which can be marked as the real structure and the “pseudo structure”, respectively. The relationships between the real structure and the pseudo one are listed as follows: (1) their stiffness matrices are identical; (2) their mass matrices are different; (3) their corresponding SMSs are the same, but for the pseudo one, the SMSs are mass-normalized with different mass matrices; (4) although they have different natural frequencies, the natural frequencies satisfy Equation (4). Therefore, the mass matrix of the pseudo structure $M_{d}$ can be obtained by the following orthogonal conditions:

(5) $$\Phi_{\varepsilon }^{T}M_{p}\Phi_{\varepsilon }=I$$

where $\Phi_{\varepsilon }$ denotes the arbitrary-scaled SMS matrix of the real structure with $\psi_{i}^{\varepsilon }$ as its modal vector, and *I* is the identity matrix. Only when the number of measured modes is equal to the measured freedom degrees does the system of equations have a unique solution. However, this condition is difficult to be satisfied. Therefore, the system of equations is often overdetermined or underdetermined, which requires the least square method, gradient projection method, etc. to solve the problem, and these methods are quite tedious. The improved approach will be proposed and discussed in Section 3.

The eigenequations of the real and pseudo structures are, respectively, obtained as follows:

(6) $$\left(K-{\bar{\omega }}_{i}^{2}M_{r}\right)\psi_{i}^{\varepsilon }=0$$

(7) $$\left(K-\omega_{i}^{2}M_{p}\right)\psi_{i}^{\varepsilon }=0$$

where $M_{r}$ is the mass matrix of the real structure, and **K** is the stiffness matrix of the real structure and the pseudo structure.

Substituting Equation (7) into Equation (6) to eliminate unknowns **K**, one can obtain:

(8) $$\left(M_{p}-\frac{1}{r_{i}^{2}}M_{r}\right)\psi_{i}^{\varepsilon }=0$$

where $r_{i}$ and $M_{r}$ are unknown and need to be solved. It should be noted that $M_{r}$ can be reasonably assumed diagonal to reduce the unknowns in Equation (8).

Supposing that the m^th^ element of the $M_{r}$ is $r_{m}$, we assume that ${\bar{M}}_{r}$ is defined as follows:

(9) $${\bar{M}}_{r}=\frac{1}{r_{m}}M_{r}$$

Substituting Equation (9) into Equation (8), and sorting out the formula as follows:

(10) $$\left(M_{p}-\frac{1}{\eta_{i}}{\bar{M}}_{r}\right)\psi_{i}^{\varepsilon }=0$$

where

(11) $$\eta_{i}=r_{i}^{2}r_{m}$$

Then, a unique solution for $\eta_{i}$ and ${\bar{M}}_{r}$ is estimated for each arbitrary-scaled SMS vector $\psi_{i}^{\varepsilon }$ by solving Equation (10). Although $\eta_{i}$ can be obtained by the above procedure, the mass normalization factor $r_{i}$ cannot be identified through Equation (11). However, the ratio $\gamma_{i}$ between $r_{i}^{2}$ and $r_{1}^{2}$ can be equivalent to that of $\eta_{i}$ and $\eta_{1}$.

(12) $$\gamma_{i}=\frac{r_{i}^{2}}{r_{1}^{2}}=\frac{\eta_{i}}{\eta_{1}}$$

Then SFM in Equation (3) can be rewritten as follows:

(13) $$F^{\varepsilon }=\frac{1}{r_{1}^{2}}\underset{i=1}{\overset{n}{\sum }}\frac{1}{\gamma_{i}{\bar{\omega }}_{i}^{2}}\psi_{i}^{\varepsilon }{\psi_{i}^{\varepsilon }}^{T}=\frac{1}{r_{1}^{2}}F_{P}^{\varepsilon }$$

The PSFM $F_{P}^{\varepsilon }$ can be obtained in the following way:

(14) $$F_{P}^{\varepsilon }=\underset{i=1}{\overset{n}{\sum }}\frac{1}{\gamma_{i}{\bar{\omega }}_{i}^{2}}\psi_{i}^{\varepsilon }{\psi_{i}^{\varepsilon }}^{T}$$

Regardless of the SMSs scaled in tests, $F_{P}^{\varepsilon }$ in Equation (14) is proportional to the real SFM $F^{\varepsilon }$, and the scalar multiplier is $r_{1}^{2}$.

### 2.2. Definition of the Uniform Load Strain Field

According to the discussion mentioned above, SFM of the structure can be approximated as follows:

(15) $$F^{\varepsilon }=\left[f_{k,l}^{\varepsilon }\right]=\underset{i=1}{\overset{m}{\sum }}\frac{{\bar{\psi }}_{i}^{\varepsilon }{{\bar{\psi }}_{i}^{\varepsilon }}^{T}}{{\bar{\omega }}_{i}^{2}}$$

where *m* is the modal orders taken into calculation; $f_{k,l}^{\varepsilon }$, the element of PSFM, denotes the strain at the *k*th element under the unit load at the *l*th node. Then, it can be expressed as the superposition of two related mass-normalized SMS coefficients for each available mode:

(16) $$f_{k,l}^{\varepsilon }=\underset{i=1}{\overset{m}{\sum }}\frac{{\bar{\psi }}_{i}^{\varepsilon }\left(k\right){{\bar{\psi }}_{i}^{\varepsilon }}^{T}\left(l\right)}{{\bar{\omega }}_{i}^{2}}$$

For a structure, the strain at the *k*th element $\varepsilon_{k}$ under uniform unit load exerting on *n*-DsOF of the structure can be expressed as follows:

(17) $$\varepsilon_{k}=\underset{l=1}{\overset{n}{\sum }}f_{k,l}^{\varepsilon }=\underset{i=1}{\overset{m}{\sum }}\frac{{\bar{\psi }}_{i}^{\varepsilon }\left(k\right)\sum_{l=1}^{n}{\bar{\psi }}_{i}^{\varepsilon }\left(l\right)}{{\bar{\omega }}_{i}^{2}}$$

The ULSF is defined as the strain field of the structure under a uniform unit load vector:

(18) $$Ε={\left\{\varepsilon_{1},\varepsilon_{2},\cdots ,\varepsilon_{n}\right\}}^{T}=F^{\varepsilon }\cdot L$$

where $L={\left\{1,1,\ldots ,1\right\}}_{n\times 1}^{T}$ means the uniform load exerting on *n*-DsOF of the structure. As can be seen from Equations (16) and (17), the ULSF is less sensitive to measurement noise than the SFM since the operation $\overset{n}{\underset{l=1}{\sum }}\psi_{i}^{\varepsilon }\left(l\right)$ in Equation (17) eliminates the random error at each measurement point, which makes the ULSF a potentially robust damage index for SHM.

### 2.3. ULSF Difference-Based Damage Index

From here onwards, we will focus on the damage detection with plate-like structures, which is widely used in engineering structure. The ULSF of the undamaged plate is continuous and smooth, while sharp changes in the ULSF will appear at the location nearby the fault in the damaged plate. Then the damage index ΔΕ can be defined as

(19) $$\Delta Ε=\left(F^{\varepsilon ,D}-F^{\varepsilon ,U}\right)\cdot L$$

where $F^{\varepsilon ,U}$ and $F^{\varepsilon ,D}$ denote the SFM of the structure before and after damage, respectively.

To address the problem that SFMs are not available in output-only cases, $F^{\varepsilon ,U}$ and $F^{\varepsilon ,D}$ are replaced by $F_{p}^{\varepsilon ,U}$ and $F_{p}^{\varepsilon ,D}$, respectively, and the damage index can be rewritten as follows:

(20) $$\Delta Ε=\frac{1}{r_{1U}^{2}}\left(F_{P}^{\varepsilon ,U}-\frac{r_{1U}^{2}}{r_{1D}^{2}}F_{P}^{\varepsilon ,D}\right)\cdot L=\frac{1}{r_{1U}^{2}}\Delta {Ε}_{P}$$

where $r_{1U}^{2}$ and $r_{1D}^{2}$ are the first modal masses of the intact and damaged structures, respectively. It is obvious that $\Delta {Ε}_{P}$ is within a multiple of $r_{1U}^{2}$ to ΔΕ, and $\Delta {Ε}_{P}$ has the same effect as ΔΕ on damage identification. A positive value of the element in $\Delta {Ε}_{P}$ is thus assumed to be a symptom of a local decrease in stiffness associated with the occurrence of damage. Based on this assumption, the following conditions will be assumed to define the damage index *IDI*(x,y) at the location (x,y):

(21) $$\{\begin{array}{c} IDI\left(x,y\right)=\Delta {Ε}_{P}\left(x,y\right)\;if\;\Delta {Ε}_{P}\left(x,y\right)\geq 0\\ IDI\left(x,y\right)=0\;if\;\Delta {Ε}_{P}\left(x,y\right)<0 \end{array}$$

The damage index *IDI*(x,y) is undetermined until the ratio between $r_{1U}^{2}$ and $r_{1D}^{2}$ is obtained. However, if $r_{m}$ in Equation (11) is the same for the intact and damaged structures, then $r_{1U}^{2}/r_{1D}^{2}$ can be substituted by $\eta_{1U}/\eta_{1D}$, which can be estimated through Equation (10) with the first-order SMS for both the intact and the damaged structures. There indeed exists at least one element in the mass matrix which does not change, and the normalizations of mass matrices $M_{r}$ are all made on those unchanged elements through Equation (9) in practice.

## 3. The Improved Method for Constructing PSFM

If an arbitrary mass matrix $\hat{M}$ is given, ${\hat{\psi }}_{i}^{\varepsilon }$ corresponding to $\psi_{i}^{\varepsilon }$, with the supposed mass-normalized matrix $\hat{M}$, can be obtained by

(22) $${\hat{\psi }}_{i}^{\varepsilon }=\frac{\psi_{i}^{\varepsilon }}{\sqrt{{\left(\psi_{i}^{\varepsilon }\right)}^{T}\hat{M}\psi_{i}^{\varepsilon }}}$$

To make the above formula true, ${\left(\psi_{i}^{\varepsilon }\right)}^{T}\hat{M}\psi_{i}^{\varepsilon }>0$ is required, and $\hat{M}$ can be defined as a diagonal matrix with positive values for all elements. SMS matrix ${\hat{\Phi }}_{\varepsilon }$ with ${\hat{\psi }}_{i}^{\varepsilon }$ as its modal vector approximates the mass normalization condition that ${\left({\hat{\Phi }}_{\varepsilon }\right)}^{T}\hat{M}{\hat{\Phi }}_{\varepsilon }$ is a matrix with diagonal elements equal to 1 and diagonally dominant. Since ${\hat{\psi }}_{i}^{\varepsilon }$ and $\psi_{i}^{\varepsilon }$ differ only by a proportional coefficient, ${\hat{\psi }}_{i}^{\varepsilon }$ can also be regarded as an arbitrary-scaled SMS vector. When using ${\hat{\psi }}_{i}^{\varepsilon }$ instead of $\psi_{i}^{\varepsilon }$, the mass matrix of the pseudo structure adopts $\hat{M}$. The subsequent derivation process can still be performed according to Equations (6)–(14) by simply replacing $\psi_{i}^{\varepsilon }$ and $M_{d}$ with ${\hat{\psi }}_{i}^{\varepsilon }$ and $\hat{M}$, respectively. This is the basic idea of the improved method for constructing the PSFM. The procedures of the proposed method to construct PSFM are summarized as follows:

Step 1: Provide an arbitrary mass matrix $\hat{M}$ with all diagonal elements positive;

Step 2: Compute ${\hat{\psi }}_{i}^{\varepsilon }$ according to measured SMS vector $\psi_{i}^{\varepsilon }$ through Equation (22);

Step 3: Compute *η_i_* through the following formula:

(23) $$\left(\hat{M}-\frac{1}{\eta_{i}}M_{nor}\right){\hat{\psi }}_{i}^{\varepsilon }=0$$

where $M_{nor}$ is a normalized diagonal matrix with an element equal to 1. If the first element is equal to 1, then $\eta_{i}=1/{\hat{M}}_{11}$ where ${\hat{M}}_{11}$ is the first diagonal element of $\hat{M}$.

Step 4: Compute the ratio of $\eta_{i}$ to $\eta_{1}$ through the following formula:

(24) $$\gamma_{i}=\frac{\eta_{i}}{\eta_{1}}$$

Step 5: Compute PSFM through the following formula:

(25) $$F_{P}^{\varepsilon }=\underset{i=1}{\overset{n}{\sum }}\frac{1}{\gamma_{i}{\bar{\omega }}_{i}^{2}}{\hat{\psi }}_{i}^{\varepsilon }{\left({\hat{\psi }}_{i}^{\varepsilon }\right)}^{T}$$

Compared with the improved construction methods of PSFM, the original method involves inverse analysis, which is cumbersome, while the improved method is a positive analysis process, and the computational complexity is significantly reduced. However, the effect of the value of $\hat{M}$ on the damage identification is necessary since ${\hat{\Phi }}_{\varepsilon }$ and $\hat{M}$ are not strictly orthogonal.

## 4. Results and Discussions

### 4.1. Case 1: The Simulation Model Constructed in FEM

(1) Analysis of the influence of mass matrix:

By multiplying $\hat{M}$ by an arbitrary non-zero coefficient *k*, supposing $\bar{M}=k\hat{M}$, and replacing $\hat{M}$ with $\bar{M}$, the following formula can be obtained according to Equation (22):

(26) $${\bar{\psi }}_{i}^{\varepsilon }=\frac{\psi_{i}^{\varepsilon }}{\sqrt{{\left(\psi_{i}^{\varepsilon }\right)}^{T}\hat{M}\psi_{i}^{\varepsilon }}}=\frac{\psi_{i}^{\varepsilon }}{\sqrt{{\left(\psi_{i}^{\varepsilon }\right)}^{T}k\bar{M}\psi_{i}^{\varepsilon }}}=\frac{{\hat{\psi }}_{i}^{\varepsilon }}{\sqrt{k}}$$

Replacing ${\bar{\psi }}_{i}^{\varepsilon }$ and $\bar{M}$ with ${\hat{\psi }}_{i}^{\varepsilon }$ and $\hat{M}$, respectively, Equation (23) is herein rewritten as follows:

(27) $$\left(\bar{M}-\frac{1}{{\bar{\eta }}_{i}}{\bar{M}}_{nor}\right)\bar{\psi_{i}^{\varepsilon }}=0$$

If ${\bar{M}}_{nor}$ and $M_{nor}$ are normalized in the same way, for example, setting the first element equal to 1, then ${\bar{\eta }}_{i}$ and $\eta_{i}$ have the following relationship:

(28) $${\bar{\eta }}_{i}=\frac{1}{{\bar{M}}_{11}}=\frac{1}{k{\hat{M}}_{11}}=\frac{1}{k}\eta_{i}$$

According to Equation (24), one can obtain the following formula:

(29) $$\frac{{\bar{\eta }}_{i}}{{\bar{\eta }}_{1}}=\frac{\eta_{i}}{\eta_{1}}=\gamma_{i}$$

Substituting Equations (26) and (29) into Equation (27) yields:

(30) $${\bar{F}}_{P}^{\varepsilon }=\underset{i=1}{\overset{n}{\sum }}\frac{1}{\gamma_{i}{\bar{\omega }}_{i}^{2}}{\bar{\psi }}_{i}^{\varepsilon }{\left({\bar{\psi }}_{i}^{\varepsilon }\right)}^{T}=\underset{i=1}{\overset{n}{\sum }}\frac{1}{\gamma_{i}{\bar{\omega }}_{i}^{2}}\frac{1}{k}{\hat{\psi }}_{i}^{\varepsilon }{\left({\hat{\psi }}_{i}^{\varepsilon }\right)}^{T}=\frac{1}{k}F_{P}^{\varepsilon }$$

As can be seen from the derivations mentioned above, after multiplying $\hat{M}$ by the non-zero coefficient *k*, the obtained new PSFM ${\bar{F}}_{P}^{\varepsilon }$ is 1/k times that of the original matrix $F_{P}^{\varepsilon }$, which has no effect on the damage index, thus, only the relative size relationship of the elements in $\hat{M}$ needs to be considered.

**Figure 1:**
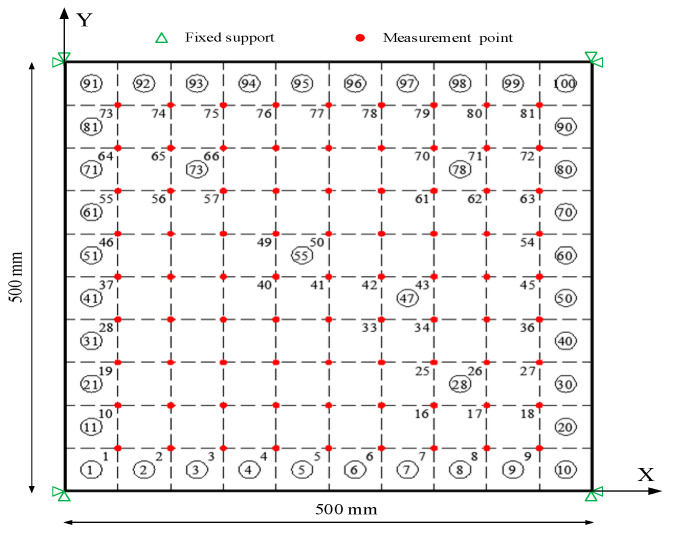
The numerical model of the plate: grid of 81 measurement points (red dots).

In what follows, a four-corner fixed plate shown in Figure 1 is considered to assess the performance of the proposed damage identification approach with different values of $\hat{M}$ under different distributions of damage and different noise-to-signal ratios. The size of the plates is 500 × 500 mm with a thickness of 2 mm. A finite element model of 10 × 10 plate elements was built in ANSYS. The plate is supported on the four corners and with free boundary conditions at all the other boundary joints. The material has a Young’s modulus in the undamaged configuration equal to 71 GPa, a Poisson’s ratio ν=0.33 and a mass density $\rho =2770\;\mathrm{kg}/m^{3}$. Due to the structural geometric and constraint symmetry, as well as the material properties being isotropic, only the strain distribution along the X-direction is considered here. Otherwise, the strain distributions along the X- and Y-directions need to be considered separately.

It is assumed that damage only affects the stiffness matrix not the mass matrix, and the damage extent is linear to the degree of reduction in Young’s modulus of the corresponding damaged element. For the supported plate, damage has been considered located around the middle span. The details of each damage case are presented in Table 1.

To study the effect of the relative relationship of each element in $\hat{M}$ on the damage identification, the following numerical experiments are performed:

Step 1: Define $\hat{M}$ to be a diagonal matrix, whose elements go to a uniformly distributed random number within the interval (1,100);

Step 2: Calculate the damage index in each case. For the convenience of comparison, the maps of damage index normalized to the maximum can be compressed into a 2D map;

Step 3: Repeat the above steps 100 times, and plot the results in a graph to observe the influence of mass matrix on damage detection.

**Figure 2:**
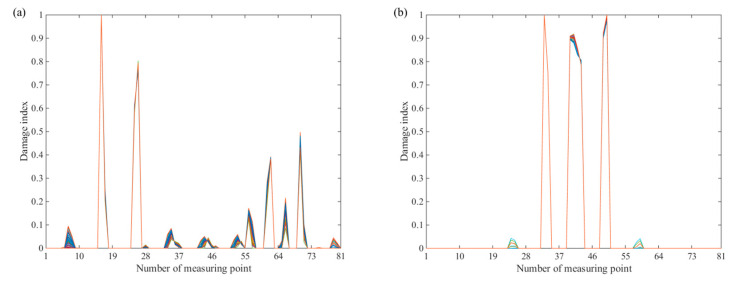
Damage index at the number of measuring points: (**a**) damage case 1 and (**b**) damage case 2.

As can be seen from Figure 2, the damages can be detected and localized by the constructed damage index under randomly distributed $\hat{M}$ matrix, which indicates that the value of $\hat{M}$ has a negligible influence on damage detection.

(2) The simplified construction method of PSFM:

As the discussions mentioned above, the matrix $\hat{M}$ can be arbitrarily given under certain conditions, thus, $\hat{M}$ can be further set to the unit diagonal matrix, and the computation steps from the Equation (26) to Equation (30) can be rewritten as follows:

(31) $${\hat{\psi }}_{i}^{\varepsilon }=\frac{\psi_{i}^{\varepsilon }}{\sqrt{{\left(\psi_{i}^{\varepsilon }\right)}^{T}I\psi_{i}^{\varepsilon }}}=\frac{\psi_{i}^{\varepsilon }}{\sqrt{{\left(\psi_{i}^{\varepsilon }\right)}^{T}\psi_{i}^{\varepsilon }}}$$

(32) $$\left(I-\frac{1}{\eta_{i}}M_{nor}\right){\hat{\psi }}_{i}^{\varepsilon }=0$$

By setting the first element of $M_{nor}$ to 1, $\eta_{i}=1/{\hat{M}}_{11}=1/I_{11}=1$, and one can obtain

(33) $$\gamma_{i}=\frac{\eta_{i}}{\eta_{1}}\;=\;1$$

(34) $$F_{P}^{\varepsilon }=\underset{i=1}{\overset{n}{\sum }}\frac{1}{\gamma_{i}{\bar{\omega }}_{i}^{2}}{\hat{\psi }}_{i}^{\varepsilon }{\left({\hat{\psi }}_{i}^{\varepsilon }\right)}^{T}=\underset{i=1}{\overset{n}{\sum }}\frac{1}{{\bar{\omega }}_{i}^{2}}{\hat{\psi }}_{i}^{\varepsilon }{\left({\hat{\psi }}_{i}^{\varepsilon }\right)}^{T}$$

By substituting Equation (31) into Equation (34), the following formula can be obtained:

(35) $$F_{P}^{\varepsilon }=\underset{i=1}{\overset{n}{\sum }}\frac{1}{{\bar{\omega }}_{i}^{2}}\frac{\psi_{i}^{\varepsilon }{\left(\psi_{i}^{\varepsilon }\right)}^{T}}{{\left(\psi_{i}^{\varepsilon }\right)}^{T}\psi_{i}^{\varepsilon }}$$

**Figure 3:**
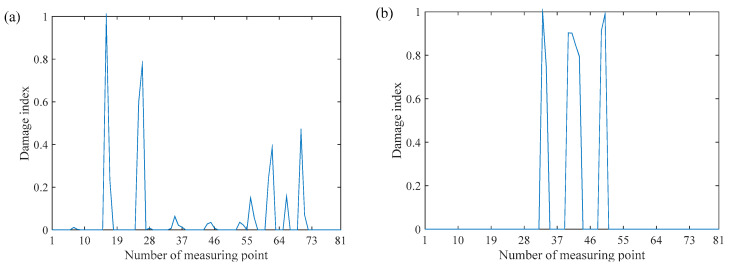
Damage detection results: (**a**) damage case 1 and (**b**) damage case 2.

Equation (35) is a simplified construction formula of the PSFM. This formula only needs natural frequencies and arbitrary-scaled SMSs, reducing the intermediate steps and being convenient for SHM. However, If the damage is so severe that it changes the mass matrix, the simplification of the method is unreasonable The calculated damage index based on the Equation (35) for the case 1 and case 2 is shown in Figure 3. In Figure 3, the peaks of damage index are correctly located at the nodes of the damaged elements. Equation (35) is only slightly different from the classic SFM, but it can be applied to the cases where the SMSs do not satisfy the mass normalization, and it is more practical.

(3) Effect of measurement noise:

According to Equation (35), the PSFM can be estimated from natural frequencies and arbitrary-scaled SMSs, which are liable to be contaminated with measurement noise in practice. To evaluate the robustness of the proposed damage detection method, the first six natural frequencies and SMSs of the numerical model of the plate have been corrupted with a random noise as follows [11,23]:

(36) $${\bar{\omega }}_{i}^{noisy}={\bar{\omega }}_{i}(1+p_{\omega }\left(2\times rand\left[0,1\right]-1\right)$$

(37) $${\left(\psi_{i}^{\varepsilon }\right)}^{noisy}=\psi_{i}^{\varepsilon }\left(1+p_{\psi }r\right)$$

where ${\bar{\omega }}_{i}$ and $\psi_{i}^{\varepsilon }$ are *i*th-simulated natural frequency and SMS without the effects of noise, respectively; $p_{\omega }$ and $p_{\psi }$ are the noise-to-signal ratio applied on natural frequencies and SMSs, respectively; ***r*** is a vector of uniformly distributed random numbers within the interval [−1, 1]. In the application of Equations (36) and (37), the parameter $p_{\omega }$ and $p_{\psi }$ are assumed equal to 1% and 5%, respectively.

**Figure 4:**
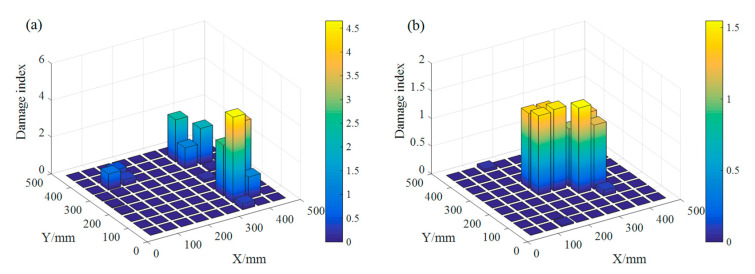
Damage index map with random noise: (**a**) damage case 1 and (**b**) damage case 2.

The measurement noise effect on different distributed damage is studied, and the results are shown in Figure 4. The relative height of the peaks corresponds to the reduction of stiffness: a higher peak corresponds to more severe damage. It is clear that the damage index based on simplified PSFM could dramatically indicate the position and degree of each damage under the influence of measurement noise.

### 4.2. Case 2: Experimental Model

**Figure 5:**
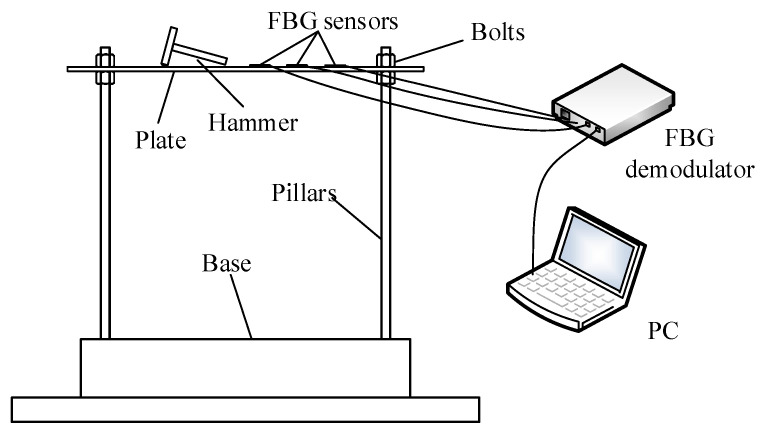
Setup of the experimental system.

**Figure 6:**
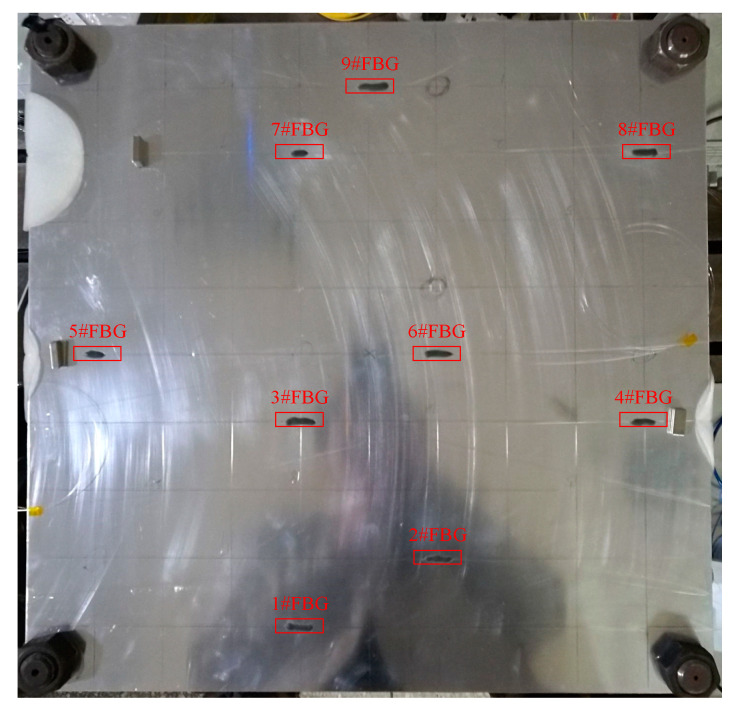
Experimental FBG sensor placement.

A plate with the fixed corner is constructed in the experiments to validate the proposed method. The setup of the experimental system is shown in Figure 5. The hammer tests on three aluminum plate specimens, respectively, named A, B, and C with the same dimensions of 500 × 500 × 2.38 mm are employed to identify the modal parameters. During the test, the instrumented hammer is used to excite the structure. However, the force signal of the instrumented hammer is not acquired since the method of the manuscript highlights the output-only damage detection. The basic material properties of the plates are all as follows: Young’s modulus E = 71 GPa; mass density $\rho =2770\;\mathrm{kg}/m^{3}$. Figure 5 shows the experimental setup for testing. Figure 6 shows the experimental Fiber Bragg grating (FBG) sensor location determined by the optimal sensor placement method [25]; the details of the optimal algorithm are shown in Appendix A. To facilitate the discussion of the results, the sensors are numbered sequentially as shown in Figure 6; the coordinate system is similar to that of Figure 1. The FBG demodulator with sampling frequency of 2 kHz is used to acquire the signals of the FBG sensors, and each time acquisition length is 5 s.

**Figure 7:**
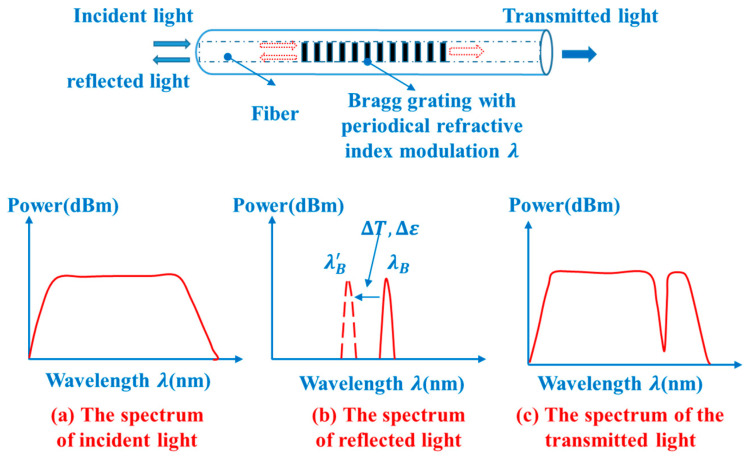
The schematic illustration of working principle of an FBG sensor.

The schematic illustration of working principle of the FBG sensor is shown in Figure 7.

As is shown in Figure 7, the broad band light will be reflected as narrow band light when it enters into the fiber and meets the Bragg condition. The wavelength of the reflected light can be determined by

(38) $$\lambda_{B}=2n_{eff}\cdot \Lambda$$

where $n_{eff}$ is the core index of refraction, and Λ is the Bragg grating period of index modulation. In addition, the parameters $n_{eff}$ and Λ are dependent on the strain and temperature, thus causing a shift in the wavelength.

(39) $$\frac{\Delta \lambda_{B}}{\lambda_{B}}=\left(1-p_{e}\right)\cdot ∆\varepsilon +\left(\alpha +\xi \right)\cdot ∆T$$

where $p_{e}$ is the elastic optical coefficient, α is the thermal expansion coefficient of the fiber, ξ is the thermos-optic coefficient of the fiber, ∆ε is the variation of the strain on the grating, ΔT is the variation of the temperature on the grating, and $\Delta \lambda_{B}$ is the variation of the wavelength of the reflected light.

The variation of the wavelength of the reflected light is induced by the temperature and the strain of the measured structure simultaneously. However, the influence of the temperature on the signal of the reflected light is static, and the strain during the test is dynamic. Therefore, during the analyzation, the *detrend ()* function in MATLAB is used to eliminate the influence of temperature on the FBG sensors. Then, the dynamic strain signal at the measuring points can be obtained by the following formula:

(40) $$\frac{∆\lambda_{B}}{\lambda_{B}}=\left(1-p_{e}\right)\cdot \Delta \varepsilon$$

**Figure 8:**
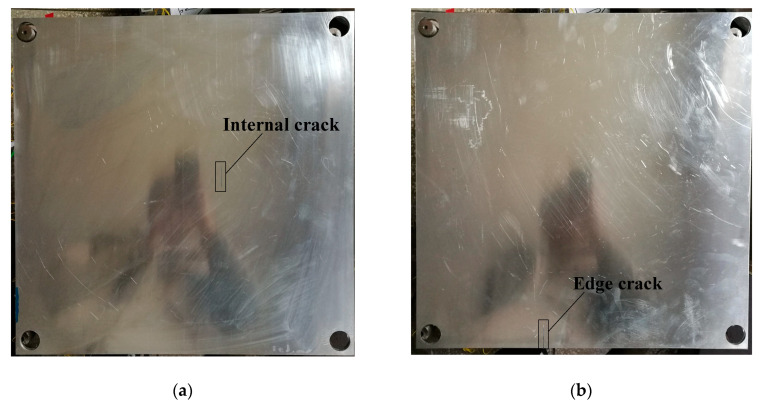
The distribution of artificial cracks: (**a**) Plate B, (**b**) Plate C

The crack damage is used to verify the proposed damage detection method herein. As shown in Figure 8a,b, the cracks are made by laser cutting technology in plate B and C, while plate A remains in the intact state as the baseline. The position coordinates of the damage are listed in Table 2.

**Figure 9:**
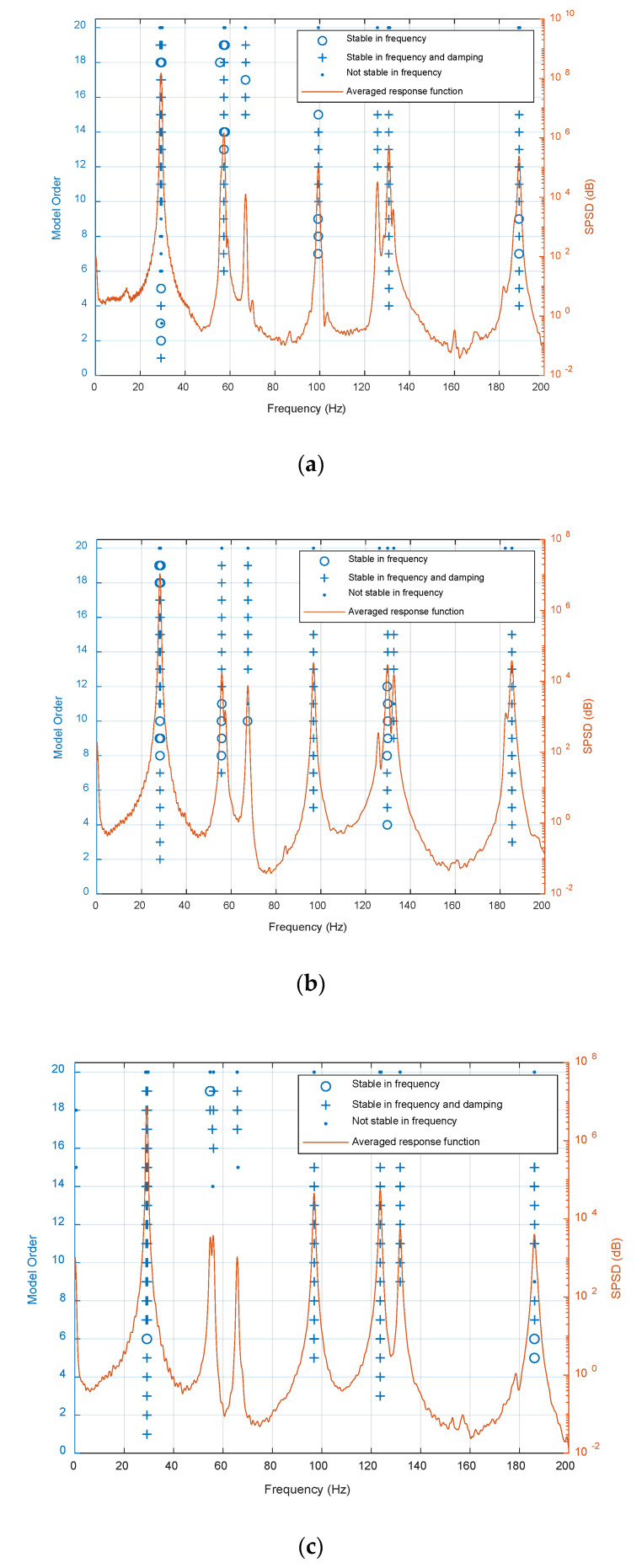
Stabilization diagrams of (**a**) plate A, (**b**) plate B and (**c**) plate C.

Firstly, the sum of strain power spectral density (SPSD) estimated from the FBG sensors on each plate specimen is used to form the stabilization diagrams shown in Figure 9; the employed modal parameter identification method is from our previous work, in which the least squares complex frequency domain method and transmissibility are combined to identify the natural frequencies and SMSs [10]. At the physical poles, the natural frequencies, damping ratios, and SMSs are unchanged with respect to the polynomial order $N_{m}$ used in the fitted polynomial for modal parameters identification in our previous study work [10]. If the variations of the natural frequencies, damping ratios, and SMSs with respect to the model order are less than a pre-set threshold simultaneously, a stable pole will be produced. In this paper, the natural frequencies and the damping are used to be the stable criterion simultaneously. Secondly, based on the stabilization diagrams, the first six natural frequencies and SMSs are estimated and listed in Table 3. Thirdly, the PSFMs can be constructed based on the obtained natural frequencies and non-mass normalized SMSs utilizing the proposed simplified calculation formulas. Finally, the PSFM-based ULSF of the intact and damaged plates is computed, and the damage index map for the plate B and plate C with the ULSF considering plate A as the baseline is formed.

To locate damage more reliably, the threshold of $∆E_{p}$ can be estimated in terms of the average $\mu_{∆E_{p}}$ and the variance $\sigma_{∆E_{p}}$ of the values of $∆E_{p}$ at all the instrumented locations. The damage threshold $∆E_{p}^{threshold}$ can be defined as [26]

(41) $$∆E_{p}^{threshold}=\mu_{∆E_{p}}+\nu \sigma_{∆E_{p}}$$

where ν is a variable parameter base on the accepted probability of false alarm—it is taken as 2 herein.

**Figure 10:**
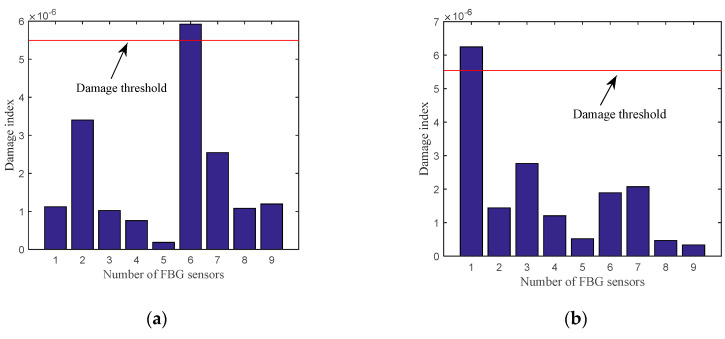
Damage indexes of (**a**) plate B and (**b**) plate C.

Figure 10a,b show the damage detection results for plate B and plate C, respectively. Figure 10 displays the damage index at the location where induced crack damage is relatively larger and above the damage threshold. From Figure 10a,b, the cracks can be detected and localized effectively.

**Figure 11:**
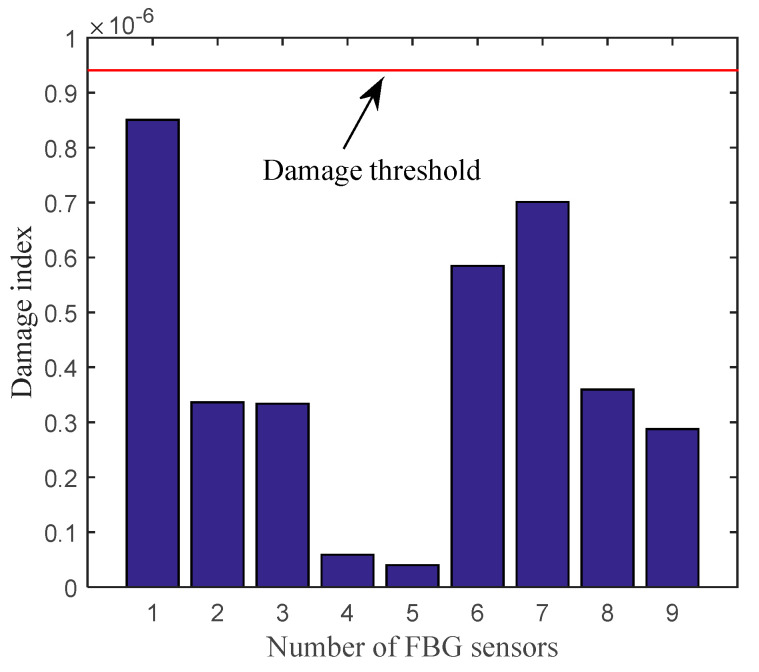
Damage index of plate A.

To verify that the damage identification method could not cause “false alarms” when the plate stays intact, a comparison test is added: the dynamic responses measured at two different times of plate A are processed separately—one is used as intact and the other is to be assessed. The damage detection results are shown in Figure 11. As can be seen from Figure 11, when the damage detection method is applied to the same intact state of the plate, the damage indices at the position of FBG sensors are all below the damage threshold of 9.41 × 10^−7^.

## 5. Conclusions

In this paper, a novel PSFM-based damage detection method is proposed to develop the SFM method for SHM in the output-only cases. Firstly, the PSFM based on the natural frequencies and arbitrary-scaled SMSs is derived. Then, the ULSF method is introduced, and the damage index based on the difference of ULSF integrated with PSFM is defined to detect damage in plate-like structures. Secondly, an improved construction procedure for PSFM is developed to reduce the intermediate steps and is verified numerically. Experimental tests have also been carried out on three aluminum plates to verify the proposed method. The experimental results showed that the proposed method is effective at detecting and localizing damage by using output-only dynamic strain information. The acceptable accuracy in damage localization is desired with a lower computational effort. However, it should be noted that the present method is limited to single crack detection, localization and the detection-relative damage degree. The errors in the present method may come from the process of modal parameter identification or the instruments in the test. In future work, a correlation between the possibility of obtaining false alarms and the redundancy of sensors will be further studied.

## Figures and Tables

**Table 1:** Damage cases in numerical analysis.

Case	Element N.	Damage Extent
1	64	15%
	73	20%
	28	30%
	18	40%
2	36	25%
	38	20%
	47	25%

**Table 2:** Location of artificial cracks in the three plate specimens.

Parameters	Center (x, y) (mm)	Length (mm)	Width (mm)	State
Plate A	—	—	—	Intact
Plate B	(325, 250)	50	0.3	Damaged
Plate C	(225, 25)	50	0.3	Damaged

**Table 3:** Strain modal parameters of intact and damaged plates.

Modes	Plates	Natural Frequencies (Hz)	Modal Strain								
			1#	2#	3#	4#	5#	6#	7#	8#	9#
1st	A	29.25	−11.4	−15.9	−13.4	2.16	2.37	−12.5	−9.26	6.91	−11.7
	B	28.38	−4.06	−6.22	−6.14	0.83	0.60	−7.00	−2.81	3.10	−4.42
	C	29.13	3.18	2.52	1.95	−0.39	−0.75	2.58	2.14	−1.01	1.93
2nd	A	57.25	1.16	1.35	0.16	−0.18	−0.04	0.16	−1.25	0.88	−1.69
	B	57.13	6.40	5.61	2.40	−0.69	−0.01	0.08	−3.37	3.83	−8.22
	C	54.88	5.49	4.51	1.99	−0.60	0.11	0.50	−2.39	2.40	−5.03
3rd	A	67.0	−1.12	−1.26	−1.08	0.05	−0.23	−1.15	−1.01	0.50	−1.70
	B	67.13	3.24	3.03	3.01	−0.05	0.46	2.66	2.30	−1.31	5.02
	C	65.88	0.64	0.55	0.59	0.04	0.14	0.52	0.58	−0.32	1.26
4th	A	99.38	1.15	−0.16	−2.18	0.19	0.10	−2.33	0.01	−0.58	1.09
	B	97.13	−0.66	0.31	1.46	−0.06	−0.06	1.97	−0.20	0.58	−1.34
	C	96.88	−0.53	0.30	0.6	−0.16	−0.04	1.16	−0.02	0.09	−0.13
5th	A	125.8	1.52	−1.84	0.94	−0.14	0.26	−0.69	−0.43	−0.43	−0.12
	B	125.6	−2.54	2.48	−1.72	0.17	−0.32	1.11	0.58	0.61	0.31
	C	123.6	3.21	−3.41	2.17	−0.15	0.37	−1.07	−0.64	−0.55	−0.90
6th	A	130.8	−0.64	2.56	−2.18	0.56	−0.88	2.44	−2.21	−0.46	−0.71
	B	129.4	0.37	−1.59	1.73	−0.32	0.52	−1.88	0.96	0.16	0.48
	C	130.3	0.08	−1.14	0.83	−0.17	0.26	−0.73	0.23	−0.07	0.69

**Figure A1:**
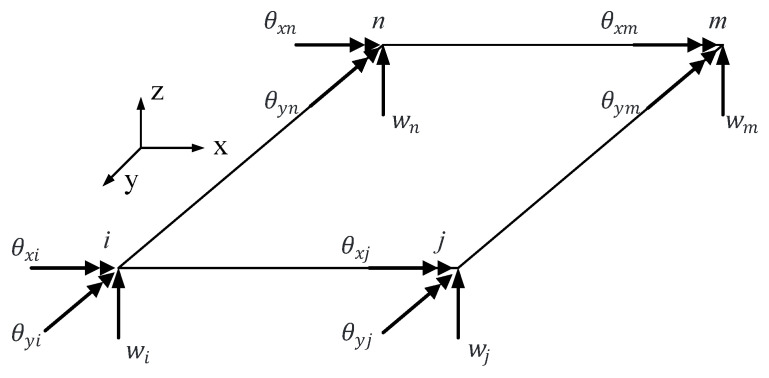
The quadrilateral element and the definition of degrees of freedom for the noses.

**Figure A2:**
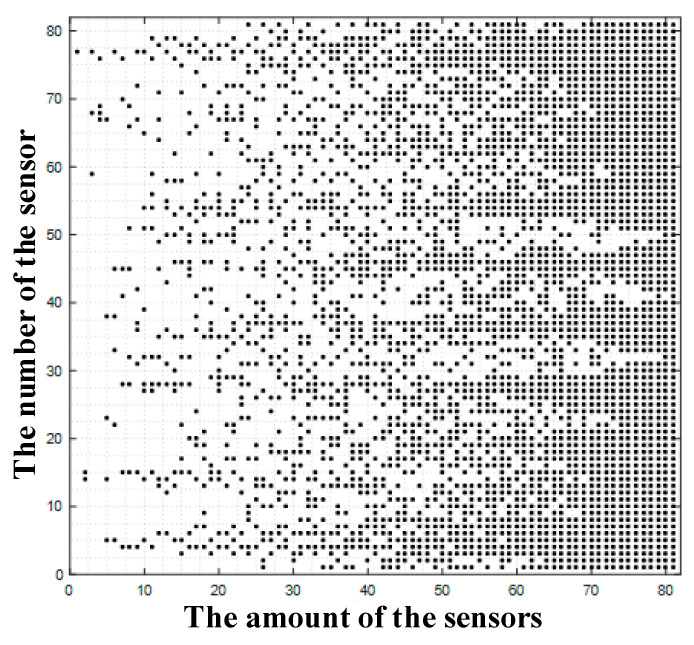
Optimal placement of the structure under different numbers of sensors.

## Appendix A

A quadrilateral element with four nodes, i.e., i, j, m and n, is used to form the FEM the plate. The quadrilateral element and the definition of degrees of freedom for the noses are shown in Figure A1. Since only the bending vibration is considered, only the three degrees of freedom, i.e., the freedom of movement along the z-direction w, the rotational degrees of freedom along the x direction $\theta_{x}$ and the rotational degrees of freedom along the y direction $\theta_{y}$.

We assume the w can be expresses as follows:

(A1) $$\begin{array}{ll} w= & a_{1}+a_{2}x+a_{3}y+a_{4}x^{2}+a_{5}xy+a_{6}y^{2}+a_{7}x^{3}+a_{8}x^{2}y\\ & +a_{9}xy^{2}+a_{10}y^{3}+a_{11}x^{3}y+a_{12}xy^{3} \end{array}$$

where $a_{i}\left(i=1,2,\ldots ,12\right)$ is constant coefficients. Then, the $\theta_{x}$ and $\theta_{y}$ can be expressed as follows:

(A2) $$\begin{array}{c} \theta_{x}=+\frac{\partial_{w}}{\partial_{y}}=a_{3}+a_{5}x+2a_{6}y+a_{8}x^{2}+2a_{9}xy+3a_{10}y^{2}+a_{11}x^{3}+3a_{12}xy^{2}\;\\ \theta_{y}=-\frac{\partial_{w}}{\partial_{x}}=-a_{2}-2a_{4}x-a_{5}y-3a_{7}x^{2}-2a_{8}xy-a_{9}y^{2}-3a_{11}x^{2}y-a_{12}y^{3} \end{array}$$

Equations (A1) and (A2) can be expressed as in a unified form as follows:

(A3) {u}=[P]{a}

where $\left\{u\right\}={\left\{w,\theta_{x},\theta_{y}\right\}}^{T}$, $\;\left\{a\right\}={\left\{a_{1},a_{2},\cdots ,a_{12}\right\}}^{T}$ and $\left[P]=[\begin{array}{llllllllllll} 1 & x & y & x^{2} & xy & y^{2} & x^{3} & x^{2}y & xy^{2} & y^{3} & x^{3}y & xy^{3}\\ 0 & 0 & 1 & 0 & x & 2y & 0 & x^{2} & 2xy & 3y^{2} & x^{3} & 3xy^{2}\\ 0 & -1 & 0 & -2x & -y & 0 & -3x^{2} & -2xy & -y^{2} & 0 & -3x^{2y} & -y^{3} \end{array}\right]$. The displacements for the quadrilateral element can be expressed as follows:

(A4) $$\left\{q^{e}\right\}=\left[C\right]\left\{a\right\}$$

where $\left\{q^{e}\right\}={\left\{u_{i},u_{j},u_{m},u_{n}\right\}}^{T}$, $\left[C\right]={\left[P_{i},P_{j},P_{m},P_{n}\right]}^{T}$. Then, based on the Equations (A3) and (A4), the following equation can be obtained.

(A5) $$\left\{u\right\}=\left[P\right]{\left[C\right]}^{-1}\left\{q^{e}\right\}=\left[N\right]\left\{q^{e}\right\}$$

where $\left[N\right]=\left[P\right]{\left[C\right]}^{-1}$ is 3 × 12 order shape function matrix. Then, the strain of the nodes can be expressed as follows:

(A6) $${\left\{\kappa_{x},\kappa_{y},\kappa_{xy}\right\}}^{T}={\left\{-\frac{\partial^{2}w}{\partial x^{2}},-\frac{\partial^{2}w}{\partial y^{2}},-2\frac{\partial^{2}w}{\partial xy}\right\}}^{T}=\left[Q\right]\left\{a\right\}=\left[B\right]\left\{q^{e}\right\}$$

where $\left[B\right]=\left[Q\right]{\left[C\right]}^{-1}$ is a matrix with dimensions of order 3 × 12. Then, the stiffness matrix of the element can be expressed as follows:

(A7) $$\left[k\right]=\iint {\left[B\right]}^{T}\left[D\right]\left[B\right]dxdy$$

where [D] is a matrix with dimensions of 3×3. Assuming the ith element in the plate is damaged, the stiffness matrix for the damaged structure can be expressed as follows:

(A8) $$\left[K_{s}^{d}\right]=\left[K_{s}^{u}\right]-\alpha_{i}\left[K_{s}^{i}\right]$$

where $\left[K_{s}^{u}\right]$ denotes the stiffness matrix of the structure before damage, $\left[K_{s}^{i}\right]$ is the stiffness matrix for the ith element under the global coordinate system, and $\alpha_{i}$ ($0<\alpha_{i}<1$) is the damage degree coefficient for the ith element.

Based on the geometric equations of elasticity, the relationship between the strain flexibility $\left[F^{\varepsilon }\right]$ and the displacement flexibility $\left[F^{s}\right]$ can be expressed as follows:

(A9) $$\left[F^{\varepsilon }\right]=\left[\tilde{B}\right]\cdot \left[F^{s}\right]$$

where $\left[\tilde{B}\right]=\left[B\right]\cdot \left[\beta \right]$, in which [B] is the geometric matrix and [β] is the transformation matrix that converts the local coordinate system into the global coordinate system. Based on the formula in Equation (A9) and the formula, i.e., $\left[F^{s}\right]={\left[K^{s}\right]}^{-1}$, the change in the strain flexibility of the structure after damage can be expressed as follows:

(A10) $$∆\left[F^{\varepsilon }\right]=\left[\tilde{B}\right]\cdot \left\{{\left(\left[K_{s}^{u}\right]-\alpha_{i}\cdot \left[K_{s}^{i}\right]\right)}^{-1}-{\left[K_{s}^{u}\right]}^{-1}\right\}$$

Equation (A10) can be expanded by using the Riemann series as follows:

(A11) $$∆\left[F^{\varepsilon }\right]=\left[\tilde{B}\right]\cdot \left\{\left({\left[K_{s}^{u}\right]}^{-1}+\alpha_{i}\cdot {\left[K_{s}^{u}\right]}^{-1}\cdot \left[K_{s}^{i}\right]\cdot {\left[K_{s}^{u}\right]}^{-1}+\alpha_{i}^{2}\cdot {\left[K_{s}^{u}\right]}^{-1}\cdot \left[K_{s}^{i}\right]\cdot {\left[K_{s}^{u}\right]}^{-1}\cdot \left[K_{s}^{i}\right]\cdot {\left[K_{s}^{u}\right]}^{-1}\right)-{\left[K_{s}^{u}\right]}^{-1}\right\}$$

After ignoring the high-order terms in Equation (A11), we can obtain the first-order sensitivity of $\left[F^{\varepsilon }\right]$ as follows:

(A12) $$\frac{\partial \left[F^{\varepsilon }\right]}{\partial \alpha_{i}}=\left[\tilde{B}\right]\cdot {\left[K_{s}^{u}\right]}^{-1}\cdot \left[K_{s}^{i}\right]\cdot {\left[K_{s}^{u}\right]}^{-1}=\left[F_{u}^{\varepsilon }\right]\cdot \left[K_{s}^{i}\right]\cdot \left[F_{u}^{s}\right]$$

Then, $∆\left[F^{\varepsilon }\right]$ can be expressed as follows:

(A13) $$∆\left[F^{\varepsilon }\right]=\overset{n}{\underset{i=1}{\sum }}\alpha_{i}\cdot \frac{\partial \left[F^{\varepsilon }\right]}{\partial \alpha_{i}}=\left[F\left(K\right)\right]\cdot \delta A$$

where $\left[F\left(K\right)\right]=\left(\left[F_{u}^{\varepsilon }\right]\cdot \left[K_{s}^{1}\right]\cdot \left[F_{u}^{s}\right],\left[F_{u}^{\varepsilon }\right]\cdot \left[K_{s}^{2}\right]\cdot \left[F_{u}^{s}\right],\cdots ,\left[F_{u}^{\varepsilon }\right]\cdot \left[K_{s}^{n}\right]\cdot \left[F_{u}^{s}\right]\right)$, *n* is the number of damaged elements, $\delta A={\left[\alpha_{1}\cdot I,\;\alpha_{2}\cdot I,\cdots ,\alpha_{n}\cdot I\right]}^{T}$, and I is the identity matrix.

From Equation (A13), δA can be obtained as follows:

(A14) $$\delta A={\left({\left[F\left(K\right)\right]}^{T}\cdot \left[F\left(K\right)\right]\right)}^{-1}\cdot {\left[F\left(K\right)\right]}^{T}\cdot ∆\left[F^{\varepsilon }\right]$$

Now, the matrix *Q* = ${\left[F\left(K\right)\right]}^{T}\cdot \left[F\left(K\right)\right]$ is defined as the Fisher information matrix. If we use an effective unbiased estimator to maximize the matrix *Q*, the best estimate of the damage coefficient vector δA can be obtained. Different nodal degrees of freedom have different contributions to the matrix. If the nodes in the candidate sensor set contribute little to the *Q*, they should be discarded. On the contrary, the freedoms that make great contributions should be preserved. Finally, the optimal sensor network layout under a certain number of sensors is obtained. When the second norm of the matrix *Q* takes the maximum value, the best unbiased estimate of can be obtained. The second form of *Q* can be expressed as follows:

(A15) $${\left\|Q\right\|}_{2}=\|{\left[F\left(K\right)\right]}^{T}\cdot {\left[F\left(K\right)\right]\|}_{2}={\left\|\left[F\left(K\right)\right]\right\|}_{2}^{2}=\lambda_{max}\left(Q\right)$$

where $\lambda_{max}\left(Q\right)$ denotes the largest eigenvalue of *Q*. Then, the objective function can be expressed as follows:

(A16) $$f(L_{n1})=\|Q{\|}_{2}$$

where $L_{n1}$ denotes the optimal layout under the n1 measurable degrees of freedom on the structure. During the optimization, the genetic algorithm (GA) is employed. The schematic diagram of the plate during the tests is shown in Figure 1. From Figure 1, obviously, the formula $n_{1}=81$ is obtained. The optimal placement of the structure under different number of sensors is shown in Figure A2.

During the test, the amount of the FBG strain sensors is 9, and the number for the FBG strain sensors on the structure can be obtained from Figure A2.
